# Doctor Pole's Thermometrical, Barometrical, and Udiometrical Statements for the Seven Preceding Years; from Daily Observations, Made by the Assistance of Accurate Instruments Employed for That Purpose, in St. James's Square, Bristol, Jan. 15, 1811

**Published:** 1811-02

**Authors:** 


					142 Dr. Poles Meteorological Statements.
For the Medical and PhysicdH Journal.
DOCTOR POLE'S Therinometricnl, Barometrical, and Udiometrical Statements for the Seven
preceding Years; from Daily Observations, made by the assistance of accurate Instruments
employed for that purpose, in St. James's Square, Bristol, Jan* 15, 1811.
The average temperature of each month, from observations
made at eight o'clock in the morning.
Months
as deno-
minated
in the
Calendar
1804
1805
3 -
1806
ti
1307
tr
a 2
1808
3 2
18091
1810
Q 2-
q 2
Jan.
Feb-
March
\pril
May
June
July
Vugust
Sept.
Oct.
\ov"
Dec.
33 50
35 33
36 33
37 39
57 -i
G2 -
G2 -
GO 33
56 3<J
49 <JfJ
42 10
,33 50
|37
37
37
43
54
Gl
52 G3
33 G2
? 54
? ?18
? 45
-,44
i9
64
63
|48
66151
20 34
44 '31
61
Gl
56
46
36
10137
32 24
34 57
39 45
45 50
50 12
60 53
Gl 71
?'.'1 32
56 40
47 71
40 ?
38 32
i The average state of the Barometer for each month, from
observations made at eight o'clock in the morning.
Months
as deno-
minated
in the
I Calendar
1804
1805
= ? 2 o
180G
1807
1808 1809
o C y C , ?/ w v
s c ~ c s o e
1810
js
Jan.
Feb.
March
April
May
J nne
July
August
Sept.
Oct.
Nov.
Dee.
39 6<;
30 25'
29 70
29 80
29 4C
30 35
29 9C
30 ?
30 6'E
29 75
29 90
29 8?
CO 29
35 '50
-29
90 29
? .">o
5 30
-'2.9
35 30
530
95 29
30 29
80 30
0
[29
30
|30
30
30
[30
129
[29
30
129
2cl30
10
5
3 o;
15
15
25
25
45
S0!.31
1I|:0 ?
75 29 75
58i29 91
80 29 67
10 29 35
3030 ?
4229 72
49,29 80
40 29 94
4;29 82
10,29 37
13J29 18
Dr. Pole s Meteorological Statements. 143
Aii account of the quantity of rain fallen in each month.
Months
as deno-
minated
in the
Calen-
dar.
180-i
1805
Jan.
Feb.
March
{April
[May. "
Tune
July
(August ; 2
ISepr.
|Ocl..
NoV.
I )ec.
mti
;1807
1808
QJ Q
"v o
2 O
i8oy
a o
V C
s ?
1810
S O
3 8?
4 27
1 81
1 49
3 36
6 as
2 28
2 1
0 34
0 4fi
5 82
0 J7>
4 -2l
2 53
3 69
2 14
5 44
2 5
1 5
0 59
0 35
5 37
99
75
76
6
52
4 12
3 26
1
3 75
1 45
1 75
1 7
4 38
4 16
0 8
1 54
2 63
0 90
2 30
0 62
1 42
2 59
1 55
4 52
2 66
2 66
3 45
6 80
5 24
?Total in?atsh year 2y 77|26 Ip4 3^31 31|38 8f29 5l]35 71
The highest temperature of the Atmosphere indicated by
the Thermometer, at any one time during the last
Seven Years.
2 S
e =
Months
as denominated
in the
Calendar.
Years.
June
July
September
June
July
July
July
September
25
ir
18
10
22
12
27
2
86
en
77
84
85
91
78
80
-1804
1805
1 SOS
1807
1808
180.9
1810
Ul
Dr. Poles Meteorological Statements.
The quantity of rain fallen in each month, upon an
average of the last Seven Years.
3 c-
*5 3
9
10
11
U
Months,
as denomi-
nated in the
Calendar.
January
February
March
April
May
June
July
August1
September
October
November
December
The lowest temperature of the Atmosphere, indicated
by the Thermometer, on the two coldest days in
the last Seven Years, according to observa-
tions made at eight o'clock m the
morning.
II
Months,
as denomi-
nated in the
Calendar.
Q
Years.
3
12
2
11
1
10
.1
12
1
12
1
11
2
12
March
December
February
November
January.
October
January .
December.
January
December
January
November
February
December
23
24&30
2
21
30
24
? 15
.23
22
21
23
20
18'
31
20
21
23
18
20
29
18
1G
12
19
10
16
16
21
Correct statements of Atmospherical changes in tempera-
ture, gravity, humidity and dryness, quantity of rain, falls
of snow, direction, force, and continuance of winds, when
comprehending a long series of time, may be expected to de-
velope some principle, or demonstrate some state of the air
connected with widely spreading epidemics. The physicians
of former periods, from Hippocrates to Sydenham, very
uniformly
Observations on Meteorological Registers. 145
Uniformly fell into this opinion. But as tliey wanted instru-
ments, their observations had a confined range, were vaguely
made, and consequently indeterminate. In their details the
seasons are distinguished as very hot, dry, cold, or wet: the
summers are described as unusually cold, or oppressive from
I'eat; the winters arc characterized by the lcyig or short dura-
tion of frost. It was not possible to shew the precise degree
of any of these. From the want of meteorological records,
it is still disputed what changes have taken place in .-the cli-
mate of the British islands, if we were to admit the. state-
ments of some of our old historians, who, in pages of flowery
eloquence, describe the genial warmth of the British vallies,
"where even the vine flourished with luxuriance, and pro-
duced the richest grapes, it must be allowed that the tempe-
rature of this country has been considerably lowered. Butap.
observation has been made by an ingenious naturalist (p. 97 of
this Number), from a nearly demonstrable fact, that in some
very early period, these regions, the'ultima thule of the Ro-
mans, were much colder than at present. If in these early
periods it had been possible to have kept correct registers of
the weather, and such registers had been continued from age
to age, they would, it is probable, have afforded data ex-
plaining operations of the atmosphere upon animal life,
which now remain in obscurity. Let us bear in mind that
"we possess the means of delivering down to future ages me-
teorological facts, which may prevent our successors exper-
iencing difficulties with regard to the present which we now
feel of the past time.
Of all the diseases which appear to be diffused by the at-
mospherical medium, the epidemical catarrh, under the
popular term Irifluenza, has the strongest character, and the
widest range, if we could know the exact state of the at-
mosphere, in all its bearings, of 1510, the earliest recorded
instance of this epidemic, and in the subsequent times of its
Recurrence in 1557, 1.580, 1587, 1591, 1675, 1709, 1733,
1743, 176?, 1767; 1775, and 1782, there would be a pro-
bability of arriving at some principle explanatory of its
cause, and of its connection with or dependence on some state
?f the air. These observations have been suggested by the
preceding statement of Dr. Pole, the correctness of which
>ye cannot doubt; and to its utility we are perfectly alive.
As far, however, as these meteorological statements regard
*he practice of medicine, we will suggest that they would be
greatly improved by adding to them a column, containing a
Agister of the prevailing diseases, or such trains of morbid
actions as constitute epidemics, in each year or subdivision of
the year.
(No. 144.) V To
To (lie naturalist, as well as the physician, it would ren-
der these registers more distinct and perspicuous, if the par-
ticular kinds of instruments employed were described. ?i>-

				

